# Two worlds, one goal: a qualitative study of academics’ perceptions of student-athletes

**DOI:** 10.3389/fspor.2026.1835408

**Published:** 2026-07-14

**Authors:** Andrew Decelis, Adele Muscat

**Affiliations:** Institute for Physical Education and Sport, University of Malta, Msida, Malta

**Keywords:** academics, dual-career, sport, support, university

## Abstract

**Introduction:**

This study investigates the challenges faced by university academics (lecturing staff) in supporting student-athletes, with particular attention to the context of small island nations where sporting pathways are still evolving. It also explores their views and awareness of existing support structures, as well as their recommendations for improving institutional support.

**Method:**

Semi-structured interviews were conducted with 12 university academics across 11 faculties in Malta. Participants were selected using purposive, convenience, and snowball sampling. Data were analysed using reflexive thematic analysis to explore the range of perceived challenges and support needs.

**Results:**

The study revealed that academics faced multiple challenges in supporting student-athletes, including unawareness of student-athletes in their classes, difficulty balancing academic requirements with student-athletes' needs, mental health concerns, and cultural and generational differences. Academics recommended enhancing support systems through increased inter-faculty dialogue, targeted training, incentives for sport mentors, greater university support, a balance of student-athletes' rights and obligations, and the promotion of the student-athlete support programme.

**Discussion:**

Findings underscore the necessity of culturally sensitive, adaptable, and integrated frameworks to accommodate both athletic and academic demands. The research highlights the importance of enhanced support for academics and student-athletes in psychologically informed environments, particularly in small nations such as Malta, where unique cultural and sporting constraints exist. These insights offer valuable contributions to both theory and practice in the development of sport and education policy and support.

## Introduction

The Dual Career (DC), defined as a career focusing on sport and education or work (1), has seen a significant increase in research over the past two decades. This surge followed the European Commission (EC) White Paper on Sport (2), which introduced the term DC and highlighted the need for stakeholders to support elite athletes combining sport with education or employment. Further underscoring this importance, the European Union's (EU) Guidelines on DCs of Athletes (2012) laid the groundwork for numerous Erasmus + initiatives, with considerable European economic resources being invested in such initiatives (3). These initiatives fostered a pan-European discourse on DCs, described as a “shared body of DC knowledge providing DC stakeholders in Europe with common grounds to understand each other, communicate and cooperate on different levels” (4, p.74).

Stambulova and colleagues (2024), in the European Federation of Sport Psychology (FEPSAC) Position Statement, synthesize recent DC studies to propose postulates to promote excellence. Despite increased attention and policy support, student-athletes continue to face numerous challenges, particularly in small countries with underdeveloped sporting cultures and limited professional sport opportunities, such as Malta (5). Whilst attempts have been made to address the challenges student-athletes face, research on the challenges academics face when trying to support student-athletes is very limited—a gap this study hopes to address.

Supporting student-athletes’ education during their peak competitive years depends significantly on collaborative efforts among various stakeholders. This necessitates a well-structured support system that encompasses DC programs and appropriate legislation (6, 7), as well as psychologically informed environments (8). Various DC support programs have been developed globally to assist student-athletes in preparing for life after their sporting careers (1, 9). An analysis of DC systems across EU member states reveals diverse approaches, leading to a DC quality framework with four key dimensions: the student-athlete's entourage, relevant stakeholders, national governments, and the EU (7). Sport-friendly universities are recognized as crucial DC Development Environments (DCDEs) which strive to support athletes in balancing their elite sporting endeavors with academic or professional pursuits (10).

While academic institutions are vital for DC development, inconsistent policy implementation often leaves support dependent on individual staff attitudes. While student-athletes struggle with inflexible course delivery and prolonged absences—frequently prioritizing sport as a result (11)—Geraniosova and Ronkainen (12) argue that successful DC integration requires a deeper understanding of the social institutions that shape athletes’ behavior. Consequently, DC policies must be tailored to a specific cultural and sports context. This is especially pertinent in resource-limited, smaller nations.

### Theoretical framework

This study adopts a holistic and ecological approach to understanding DCs in sport, drawing primarily on the Holistic Athletic Career (HAC) Model (13, 14) and Ecological Systems Theory (EST) as applied to athletic development (15, 16). Together, these frameworks provide a comprehensive lens for examining university academics’ perceptions of student-athletes and the support structures surrounding them. The HAC model conceptualizes DCs as a dynamic process shaped by the interaction of multiple developmental domains—athletic, academic/vocational, psychological, psychosocial, and financial—across life stages (14). This perspective is particularly relevant in higher education contexts, as it emphasizes that successful DC development relies not only on student-athletes’ individual capacities but also on the coherence, awareness, and responsiveness of institutional support systems, including academics’ roles within these systems.

EST further situates academics’ perceptions and practices within interconnected social and institutional systems (15). At the microsystem level, everyday interactions between academics and student-athletes influence flexibility, understanding, and academic arrangements. The mesosystem comprises collaboration between faculties, sport services, and student-athlete support programs, while the exosystem and macrosystem reflect institutional policies, national sport culture, and broader societal attitudes toward sport and education (16). This ecological framing is especially pertinent in small-island contexts where limited sporting infrastructure and opportunities for professional sport careers, and close-knit academic environments, may intensify both constraints and opportunities for DC support. Taken together, these theoretical perspectives enable a multi-level, culturally sensitive analysis of DCs that integrates individual experiences, interpersonal dynamics, and institutional structures within higher education.

### European dual-career frameworks and contextual challenges in small states

One of the most frequently cited obstacles to maintaining a DC is considerable commitment and resulting heavy workload (17). Wylleman et al. (18) identify challenges student-athletes face across six developmental levels: athletic, psychological, psychosocial, academic/vocational, financial, and legal. These challenges are particularly pronounced in small states where pursuing a sporting career is often more difficult and financially unsustainable (5) due to a lack of sporting culture and professionalism (19).

FEPSAC's position statement emphasizes the need for student-athletes to manage simultaneous demands across various life domains and develop effective prioritization strategies (20). Academic support can enhance individuals’ well-being (21) and may contribute to improved athletic performance. In line with this, FEPSAC has underscored the importance of fostering mental health literacy and resilience among student-athletes (20).

In the EU, most professional and elite-level sports are organized through club systems, often lacking formal educational connections, creating significant challenges for student-athletes balancing academics with high-performance sport. The European Guidelines on DCs of Athletes (3) were developed to encourage stronger collaboration between sport organizations and educational institutions, with the aim of creating culturally appropriate frameworks for the development of DCs. Notable national programs include Germany's Partner Universities of Top Sport and Spain's PROAD (Programa de Atención al Deportista de Alto Nivel) career assistance program. These guidelines emphasize that to be effective, dual-career initiatives must be tailored to the unique requirements of student-athletes, factoring in variables such as age, specific sport, athletic maturity, and socioeconomic background. Adaptations that are sensitive to local cultural realities are also required, as guidelines from larger nations may not apply to smaller nations such as Malta, which lacks a strong sporting culture (19).

### Well-being and systemic gaps

Fuchs et al. (22) highlight cross-national differences in DC systems and emphasize the need for context-specific support, particularly given the increasing mobility of student-athletes. However, it remains unclear whether student-athletes perceive university support differently across contexts and which factors shape these perceptions (23). Higher education institutions often lack a comprehensive understanding of student-athletes’ needs, while elite sport organizations may be unfamiliar with academic demands, limiting the development of effective support structures (23).

Student-athletes consistently emphasize the need for flexible class attendance, rescheduled examinations, online learning, and individualized academic programs (24, 25). Effective support requires psychologically informed environments (8), institutional flexibility aligned with the demands of elite sport (23), and sensitivity to individual readiness, expectations, and cultural context (5, 26).

Despite aspirations for academic success, many student-athletes have lower academic goals or struggle to complete their studies due to sporting demands, often resulting in reduced engagement in both curricular and co-curricular university life (1, 9). Participation in high-level sport may therefore hinder full educational engagement unless institutions provide flexible academic pathways, personalized course guidance, and adjusted assessment schedules that accommodate sport commitments (9, 27).

The role of DC Support Providers (DCSPs) in guiding student-athletes towards DC excellence has been widely recognized (28). Nonetheless, many countries, particularly small states, continue to face challenges in implementing comprehensive DC support across educational stages and into employment. EU-funded initiatives, such as ESTPORT (Developing an innovative European Sport Tutorship model for the dual career of athletes), have sought to address these gaps by promoting sport tutoring models and institutional collaboration to facilitate academic integration (29). Similarly, New Zealand's Athlete-Friendly Tertiary Network (AFTN) advocates flexible academic arrangements and access to missed learning materials; however, limited staff awareness has constrained its effectiveness in practice (11).

Educators face inherent challenges in meeting the needs of diverse student populations, and a lack of awareness of student-athletes’ circumstances can further limit effective support (11). Structural change within universities is also complex, as policy implementation requires navigating institutional systems and shifting attitudes across multiple organizational levels (11, 30).

Evidence from national contexts illustrates both progress and persistent challenges. In Sweden, national DC guidelines introduced in 2018 increased institutional support through flexible academic pathways, collaboration with sport organizations, career transition assistance, and designated DC facilitators, as well as financial support from the National Olympic Committee (6, 31). However, ineffective communication and inconsistencies between promised and delivered support led some student-athletes to disengage from academic assistance, highlighting the importance of personal resources, support, and coping strategies in sustaining a DC (32). In contrast, German vocational and educational institutions demonstrate more integrated support through structured communication, flexible scheduling, access to missed content, and formal recognition of sporting achievements (33).

### Institutional DC support in a small island state

The Maltese educational system comprises three sectors: state schools, church schools, and independent schools. State schools are funded by the government, church schools are government-funded and allocate places through a ballot system, while independent schools are fee-paying. Education is compulsory for all children between the ages of 5 and 16 years. Schooling is organised into primary education (Years 1–6) and post-primary education (Years 7–11), followed by post-secondary (sixth form 16-18 years) and tertiary education. Till the age of 16, DC support is limited, unless students attend the National Sport School (which is government run) or the Mediterranean College of Sport (a private school).

At post-secondary level, in response to ongoing DC challenges and as a result of the first author's participation in the Erasmus + ESTPORT project (29), the University Student-Athlete Support Programme (SASP) was launched in Malta in 2018. This framework appointed a sports mentor in each faculty or institute to support student-athletes and act as a liaison. The second author of this study was also appointed as a sport psychologist to support student-athletes. Despite these efforts, student-athletes still face challenges, as sports are often perceived as secondary to academics (5). Indeed, while some educational institutions lack clear policies to support DCs, others have introduced measures, such as scheduling lectures between 9.00 am and 3.00 pm, to better accommodate training demands. Moreover, while ESTPORT examined student-athletes’ perspectives, it overlooked the pivotal role of academic staff. With the intention of understanding the challenges academics face in supporting student-athletes and in the hope of improving the SASP and other programmes in other countries, the authors of this study engaged in this study with the main research questions being:

1. What are the challenges academics face when supporting student-athletes in a small island state that lacks a strong sporting mentality?
2. What improvements to institutional support for student-athletes can be identified?

## Materials and methods

This study is situated within an interpretivist–constructivist paradigm, which assumes that reality is socially constructed and that meaning is generated through individuals’ experiences and interactions within a specific cultural context and institutional roles. Consistent with the HAC Model and EST, the study recognizes that academics’ perceptions of DCs are shaped by multiple, interacting systems (e.g., individual beliefs, faculty cultures, institutional policies, and national sport contexts). A qualitative research design was therefore adopted to capture these contextually embedded perspectives and to explore the complexity of academic support for student- athletes in a small island setting.

The authors acknowledge their positionality as researchers (34), with professional experience in sport and dual-career policy, and recognize that this background informs both data interpretation and the framing of findings. They are both university academics, a position that also facilitated access, acceptance, trust, and cooperation from participants (35). Their insider knowledge of the university and its organizational structure, particularly regarding support for student-athletes, enhanced the study's depth and relevance (34).

However, insider research also requires a heightened awareness of potential biases, including the need to distinguish personal experiences, thoughts, and emotions from those of the interview participants (36). While both authors are affiliated with a university institute, they are considered outsiders to other faculties. This dual positioning may enhance objectivity and help to mitigate bias (37).

The HAC model necessitates an approach capable of capturing multiple developmental domains (academic, psychosocial, institutional), while EST requires sensitivity to interactions across micro-, meso-, exo-, and macro-levels. These theoretical commitments justify the use of in-depth qualitative methods that prioritize participants’ lived experiences and interpretations rather than quantification or causal testing.

Data were collected through semi-structured interviews, which provide flexibility to explore issues emerging across ecological levels while ensuring coverage of key domains identified in the HAC model. The interview guide was informed by both frameworks and addressed topics such as academic–athletic balance, institutional support, inter-faculty communication, cultural attitudes toward sport, and perceived challenges in supporting student-athletes. This approach enabled participants to reflect on both individual experiences and systemic influences. The interview questions were informed by existing literature and the authors’ professional knowledge and experience in the field.

### Participant recruitment

Participants were recruited using a combination of purposeful, convenience, and snowball sampling strategies, which aligns with the ecological framework by ensuring representation across different faculties and institutional contexts. The recruitment criteria was simply that individuals needed to be an academic at the University of Malta. Initially, an email invitation was sent to all faculties at the University of Malta, a public University and the largest University on the island, inviting potential participants to contact the authors if they were willing to participate in the study. The email included an information sheet outlining the study's purpose and procedures, as well as a consent form. Efforts were made to include at least one academic from each field of study. The authors also contacted individuals with whom they were familiar from different faculties, particularly those not initially represented, using publicly available email addresses. In several cases, these academics forwarded the invitation to colleagues within their faculty whom they believed were better positioned to address the study's questions. For instance, some referred the authors to Heads of Department, thereby ensuring that participants had extensive experience within their respective Faculties. Others directed the authors to sports mentors or members of well-being committees, who were more directly involved in supporting student-athletes. The authors did not limit participation to sports mentors and department heads. They also invited academics who may not have been directly involved in DC matters, but who nonetheless taught or interacted with DC student-athletes. This approach allowed for a more comprehensive understanding of the institutional context and academic experiences of student-athletes.

A pilot interview was conducted with an academic who had several years of experience as a department head. He was also a trusted colleague of one of the authors and was selected based on his ability to provide constructive feedback on the interview questions. Following the pilot interview and the feedback received, some minor modifications were made to the interview guide. One of the concluding questions was amended from: “How should the university/educational institutions and courses move forward regarding student-athletes, considering not just local but also foreign student-athletes who may opt to come and train in Malta?” To, “How do we support other interests outside academic work?”

Following the pilot interview, twelve academics responded to the call for participants. The data from the pilot interview were also included in the analysis, bringing the total number of participants to 12, representing 11 different Faculties (see Table 1A). Eight participants were male, and four were female. All but one participant were employed full-time. The eleven full-time academics had 1–25 years of service at the university, with a mean of 14.5 years. In qualitative research, sample size is secondary to achieving data saturation (38). This was reached at the 10th interview, however, as 12 responded to the call, we interviewed them all.

**Table 1A:** Participant table.

Pseudonym	Gender	Faculty	Approx years of academic experience at time of interview
George	M	Information technology	25
Maria	F	Built environment	12
Luke	M	Arts	17
James	M	Management and accountancy	20
Claire	F	Education	10
Owen	M	Engineering	21
Noel	M	Laws	10
Kris	M	Medicine and surgery	26
Jasmine	F	Social well-being	1
Alex	M	Science	4
Joe	M	Health sciences	15
Christina	F	Social well-being	16

### Conducting the interviews

Interviews were conducted at a mutually agreed-upon time and location on campus. One interview was conducted online via Zoom because the academic was on sabbatical at the time. Most interviews took place in either the second author's or the participant's office; however, two were conducted outdoors at tables on campus. Participants were informed of both authors role within the SASP. Interviews were conducted by the second author as this role was relatively new to her at the time of interviews and previous communication with participants was minimal, apart from the pilot interview. Interviews were held in either Maltese or English, according to participants’ preference. Due to participants’ varying schedules and commitments, interview durations ranged from 36 to 105 min, with an average length of 50 min. All interviews were audio recorded with participants’ informed consent.

### Ethical considerations

The relevant research ethics committee (The University of Malta—Faculty of Education Research Ethics Committee) granted ethical clearance for this study (Approval no. EDUC-2023-00018). Participants’ consent was obtained, and they were assured of confidentiality and the right to withdraw from the study at any time without providing a reason. In such cases, any data collected from the participant would be immediately destroyed and excluded from the analysis. To protect the participants’ identities, pseudonyms were used in place of real names.

### Data analysis

Data was analyzed using reflexive thematic analysis (39), which is compatible with an interpretivist paradigm. Reflexive thematic analysis allows for the identification of patterns of meaning while acknowledging the researchers’ active role in interpretation. This method aligns with the HAC and ecological frameworks by facilitating analysis across multiple levels (individual, interpersonal, institutional, and cultural) and by supporting a nuanced understanding of how academics construct meaning around DCs.

This method involves identifying, analyzing, and reporting patterns (themes) within qualitative data. It is well-suited to a variety of research questions, particularly those based on interview data (40). The analysis followed the six-phase approach proposed by Braun et al. (40). First, the researchers familiarized themselves with the data by reading the transcripts, where necessary translated from Maltese, and noting significant details. In the second phase, initial codes were generated by assigning labels to meaningful segments of data, thereby organizing the content into coherent patterns. These codes formed the basis for theme development, based on their relevance to the research questions.

The third phase involved constructing thematic maps to explore and refine relationships among emerging themes. During the fourth phase, themes were reviewed and refined to ensure coherence, consistency, and alignment with the research objectives. Each theme was then clearly defined and named, and thematic maps were updated accordingly. Throughout this process, the authors actively engaged in critical reflection on the data and their interpretive role, consistent with the approach's reflexive nature (39). At this stage, independent coding was carried out by a critical friend outside the institution (41) to address credibility. In the final phase, the authors produced a report that evaluated how each theme functioned independently and in relation to others. This stage involved reflecting on the research questions, reviewing the coded data, refining theme definitions, and integrating the findings with existing theory and literature in the field.

### Validity, reliability, and reflexivity

To enhance validity, participants were purposively recruited from across faculties to ensure diverse perspectives. Preference was given to academics with extensive years of experience at the University. Reliability was strengthened by conducting a pilot interview, which enabled the researchers to identify and refine problematic or ambiguous questions prior to the main data collection phase.

Reflexivity was maintained throughout the research process. The first author engaged in regular discussions with the second author at each stage of the study to ensure methodological rigor and consistency in the application of research procedures. Additionally, the interview questions were not provided to the participants in advance. This decision was made to reduce the likelihood that participants would prepare scripted responses or be influenced by external sources, thereby promoting authentic, experience-based responses and enhancing the trustworthiness of the data.

## Findings and discussion

This section presents a discussion of two interrelated key themes: (1) *Navigating demands: the challenges of supporting student-athletes,* and (2) *Cultivating a winning environment: Strategies for academic support*. Theme 1 reflects misalignment and strength across HAC layers and ecological systems, whereas Theme 2 represents adaptive responses and alignment strategies across these same layers and systems. Although presented as distinct themes, the findings are conceptually interconnected and reflect different dimensions of the same dual-career ecosystem. Subthemes are presented in italics throughout this section (Table 1B).

**Table 1B:** Thematic map of academics’ perceptions on the challenges they face when supporting DC athletes and avenues for enhancing institutional support.

Themes	Sub-themes	Codes
Navigating demands: The challenges of supporting student-athletes	Definitions, roles, and lack of awareness	Student-athlete; DC mentor SASP; DC mentor for faculty; presence or not of mentor in faculty; presence of DC athletes
	Balancing the obligations of an academic with addressing student needs	Equity; fairness; large number of students; abuse of the system; group work challenges; special arrangements; student complaints; necessity for today’s student to work; lack of support by some staff; flexibility
	Mental health concerns	Lack of awareness; long waiting list for mental health services; students shy from seeking support
	Cultural and generational challenges	Faculty culture; lack of sports mentality in Malta
		Sense of entitlement of students; need for gratitude
Cultivating a winning environment: Strategies for academic support	Initiating discussions	Influence of this study; initiating discussion with faculties on DC challenges; enhanced communication
	Training and rewards for academics	Personal development on mental health and support systems; awareness of lifestyle and challenges of student-athletes; awareness of foreign DC challenges
		Academic rewards for providing support (mentors have considerable extra work)
	Student support and encouragement	Financial; students should participate in extracurricular activities; formal recognition for sport achievements
	Creating a balance	Flexibility for all students; rights and obligations; time management; more online/part-time courses; recording of lectures; exploring management of courses; career break; rigid education system
	Promotion	DC programme; increase sorting activity on campus; improving sports and academic facilities.

The first theme comprises four subthemes: definitions of student-athletes; the roles of DC mentors; academics’ limited awareness of the SASP program; student-athlete mentors; and student-athletes. It focuses on *the challenges faced by academics in balancing their obligations with the need to address student needs*. Issues such as fairness to all students, potential abuse of the system, student complaints, and other concerns are discussed. This theme also explores the *mental health concerns* evident in today's student population. *Cultural and generational challenges*, including the absence of a sports culture in Malta and students’ sense of entitlement, are also examined.

The second theme centers on moving forward and developing strategies that academics believe may help provide better support in the future (Table 1B). This includes the need for further *discussions* on the topic, within and across faculties, *personal development for academic staff* on how to support student-athletes further, *the need for mentors to be rewarded* for the extra work they engage in, *student support and encouragement* including recognition of student-athletes’ sport achievements, *creating a balance* between the rights and obligations of student-athletes, and *promotion* of sporting activity and the SASP at the university.

### Navigating demands: the challenges of supporting student-athletes

Interviews with academics highlighted varying *definitions, roles, and a lack of awareness* regarding student-athletes and their support systems. Noel defined a student-athlete as “someone who is conducting his/her studies while at the same time undertaking sports professionally or semi-professionally, “ similar to the definition put forward by Stambulova and Wylleman (1). This definition explicitly acknowledges the concurrent demands of the academic/vocational and athletic layers of the HAC model. Conversely, George viewed a DC as meaning anything, questioning the fairness of special arrangements for student-athletes. He emphasized that although other students might seek similar treatment, such privileges may not be extended to the workplace. Within an EST framework, George's stance reflects a clash at the macrosystem level, where deeply ingrained cultural values regarding institutional egalitarianism and uniform meritocracy conflict with individualized DC support. Owen also argued that flexibility should not be limited to student-athletes, as such a restriction could be considered discriminatory. Other students, such as musicians and students from disadvantaged backgrounds, may also require support.

Two of the interviewed academics serve as student- athlete mentors, and two others serve on the faculty well-being or student-request boards. The mentor's role was generally understood as that of an advocate for the rights of student-athletes. Kris described their role as functioning as a link between the Faculty and the SASP, noting the existence of smooth internal support processes. However, George stated the mentor's support is not unconditional, asserting, “I am not here to defend you just because you are a student-athlete.”

Ryan et al. (11) noted that educators’ lack of awareness of their roles in DC support policies hinders the implementation of these policies. George, Maria, Luke, James, Noel, Joe, Christina specifically cited a lack of awareness about the SASP and mentors. Additionally, Maria, Luke, James, Claire, Jasmine and Alex were unaware of their faculty's student- athlete mentor. While student-athletes value dedicated staff for support (11), academics also need to be informed about who the mentors are and their roles.

Consistent with Rossi and Hallmann (33), most academic staff reported flexible approaches to supporting DC individuals. Lecturers typically address study-unit difficulties directly, whereas faculty management addresses complex issues such as study suspensions. However, academics face *challenges in balancing their obligations with student-athletes’ needs*, primarily due to limited awareness of student-athletes among their students. Maria noted that she typically becomes aware of a student-athlete only when notified by the faculty office that an alternative assessment method needs to be arranged. Claire emphasized that lecturers need to be aware when student-athletes are enrolled in their programs in order to understand their commitments and requirements.

While providing opportunities, Claire stated that lecturers may need to go above and beyond their academic commitments out of empathy, whilst acknowledging that academics cannot treat everyone equally. She stressed the importance of equity, stating, “In order for everyone to succeed, you have to give more opportunities to some.” She applied this principle without hesitation, recognizing that “some students require more patience and dedicated time to succeed.” However, Xerri and Muscat's (5) study in Malta found mixed and at times unsupportive faculty experiences among student-athletes. This may be particularly evident in a culture where academics are regarded as more valuable than sporting commitments.

In courses with larger enrollments, accommodating changes may be more difficult, as noted by Maria and James. Maria, Luke, Joe, and Christina also expressed concern about potential misuse of the system by student-athletes who used sports involvement as an excuse to miss lectures. Joe, however, felt that no student-athlete he encountered had exploited student- athlete privileges, emphasizing that support should not tolerate such misuse and that students should be regarded as equal. Maria stressed that there needs to be some clarity on what arrangements are expected and required, so it is not used as an excuse. George noted that he typically advises students who claim to be busy, “I was just like you and had to learn time management. I never told them, “Let us see how you can get an extension or apply to the board of studies.”” Ryan et al. (11) recommend that student-athletes be organized due to time demands, otherwise they risk failing to be successful in one or both of their DC commitments.

Challenges with special arrangements were also highlighted by George, Maria and Noel. Maria described instances in which one may not be satisfied with special arrangements, such as unexpected online assessments for Erasmus students, which led other students to ask why they are sitting for an exam in a classroom, while others are given an online assignment? James also detailed student frustration and perceived unfairness regarding the provision of online lecture access to an injured student-athlete. Thus, fairness, both perceived and practiced, is a critical consideration George, Noel, Maria, and Christina noted, and as also seen in Xerri and Muscat's (5) study.

Claire advised discretion when making special arrangements, viewing it as a lesson in accommodating others. She expressed concern that students were demanding the same arrangements as their peers, driven by a “sense of entitlement or frustration,” indicating a failure to communicate that different students have different needs. Christina emphasized the importance of fostering students’ awareness of diverse needs and the role of lecturers in supporting them, thereby promoting mutual support in future team-based environments.

George ultimately deemed it an issue of fairness. He explained that granting an extension to one student could unfairly disadvantage another who met the deadline. He stated that this is not fair, and granting extensions should not be determined by whether academics pity the student, but whether it is fair. The necessity for today's students to hold part-time jobs alongside their studies to sustain their lifestyles was a recurring subtheme (Maria, Luke, Noel, Joe). However, students, including student-athletes, must reassess priorities during peak periods (4) and may need to make difficult decisions about where to direct their attention. Thus, support across several levels (university, sports organization, national team set-up) is crucial in these moments.

However, not all staff are supportive, Maria reported, whilst Owen believed that having a sporting background fosters empathy, stating,

I have seen it from some of my colleagues; they do not get it yet. They say, ‘What is your career, academics or sports? Where will sports take you? We know where sports will take us in Malta, nowhere.’ So, in a way, I do not blame them.

Conversely, Joe argued that support is character-driven, citing a non-sporting colleague who consistently shows support. Educational support is important as it not only improves student-athletes’ well-being but also performance (42).

Academics raised concerns about the extent of flexibility. Maria stated that missing all lectures for a module might be unacceptable. In contrast, Owen and Noel noted that flexibility limits apply to part-time lecturers and in fields such as medicine, where external examiners limit rescheduling. Paradoxically, Kris, the Faculty of Medicine participant, highlighted that weekly timetable changes provide flexibility, allowing adjustments such as switching tutorial groups with sufficient notice. He stated that though this might be inconvenient for staff, it might be better than a strict no, suggesting that complex flexibility is feasible and preferable.

Academics also demonstrated a lack of awareness of student *mental health concerns*, echoing Muscat et al. (43)'s findings on football coaches’ limited mental health awareness in Malta. This consistent theme underscores the prevalence of mental health illiteracy within Malta's tightly knit community. While some academics acknowledged existing support systems, they sometimes did not fully grasp their importance. Luke noted that many academics do not know the difference between psychologists, psychiatrists, and other mental health professionals. Some are not even aware of mental health issues. So, whilst the support systems exist, I am not sure that we are aware of the importance of these systems.

Luke and Claire also highlighted long waiting lists for student mental health support, compounded by students’ reluctance to seek help (Maria and Luke). This reluctance may be amplified by student-athletes’ fear of appearing weak and being judged within a sport culture that emphasises toughness and places insufficient importance on mental health (43).

*Cultural and generational challenges* also emerged as significant. Faculty culture was identified as an influencing factor, with some faculties perceived as highly supportive, especially during assessments, while others reportedly induced student anxiety. The perception that elite sport is incompatible with certain professional paths, such as medicine, also increased pressure (Kris). Luke noted their faculty's supportive culture:

In our faculty, our culture is very supportive of students. We still require the standard from the student, but we do not have the culture that we are here to scare the student. That is something informal, as it is not written anywhere, but that is culture, and sometimes culture is more important than rules.

However, Luke and Owen felt that Malta, as a nation, has not yet internalized the idea of a sports career. Xerri and Muscat (5) highlighted Malta's limited sports culture as impeding individual success.

Another issue was a perceived sense of entitlement among students. Owen remarked, “Most of them do the bare minimum to pass. There are only around 5% who want to perform.” Whilst Joe observed that the current generation is much more self-centered than previous ones, recalling students stating that no one wants to finish at 4 pm because the student-athletes have training, work and personal matters to attend to. Intergenerational differences may lead to conflicts in organizational settings and if such conflicts are not managed well, intergenerational perceptions can bring on frustration, and inhibit performance and efficiency (44). Student-athletes need to understand that compromises are necessary; for example, leaving a lecture early rather than missing it entirely. Joe also felt student-athletes should show gratitude:

You are not entitled to it, but we are giving it to you. When you say ‘thank you’ to the lecturers, they will appreciate it, and when the next student comes along, they will be more willing to help; that is life.

From an HAC perspective (13), academics’ accounts above reveal that challenges emerge when developmental domains are addressed in isolation rather than as interconnected processes. This theme illustrates tensions between HAC development domains, for example:

- Academic–athletic conflict → misalignment between academic/vocational and athletic layers
- Mental health concerns → strain within the psychological domain
- Unawareness of DC status → breakdown in psychosocial and institutional support layers
- Generational and cultural differences → disruption in value alignment across domains

These challenges highlight how misalignment across ecological levels constrains academics’ capacity to support student-athletes. These can be classified as ecological barriers at various systems:

- Microsystem: strained academic–student interactions
- Mesosystem: weak communication between faculties and sport services
- Exosystem: limited institutional recognition or incentives
- Macrosystem: national culture that undervalues elite sport.

### Cultivating a winning environment: strategies for academic support

Interviewees reported that their involvement increased their awareness of the circumstances surrounding student-athletes and that the study results will contribute to greater understanding and awareness on campus. Several academics promised to *initiate discussions* within their faculties on the challenges faced by student-athletes and the support systems available to them. Maria noted that she will raise this to the wellbeing committee to see how they can improve it. Enhanced communication and collaboration between departments is recommended, as noted by Luke and Alex.

Joe, a former student-athlete, reflected on the progress the University has made in supporting student-athletes since his time. This aligns with observations by Fahrner and Burk (23), who reported that, in the United States, the quality of university services supporting student-athletes plays a significant role in attracting them to specific institutions. In light of this, it is in the university's interest, as well as that of other universities, to continue strengthening support mechanisms for both student-athletes and academic staff, thereby promoting a more inclusive and accommodating educational environment. Wagstaff and Quartiroli (8), in fact, speak about the need to adopt a systems-led approach where psychological concepts, such as in this case, the support to be provided to student-athletes, are understood and developed by various stakeholders and not only by psychology practitioners.

Traditional academic training does not adequately prepare lecturers to address the complex and evolving needs of students, particularly student-athletes who are balancing academic and athletic commitments. For Alex, academics are not well-trained to manage stressful psychological situations, a deficit that directly threatens the student-athletes’ HAC psychological layer when academic staff fail to recognize or mitigate burnout. Greater awareness of the lifestyle and challenges faced by student-athletes is necessary (Luke, Jasmine). The participants offered several recommendations to enhance the university's support for student-athletes. Supporting student-athletes contributes significantly to their overall well-being (21).

One key suggestion was the provision of *personal development training* on mental health issues and available support systems, particularly for academic staff. Luke and Claire suggested that such training be made compulsory, similar to the professional development required for academic progression. Claire believes that there should be a moral and philosophical dialogue about what it means to be an educator. She stressed that supporting students is not an act of charity or a favor; but that's what you should be doing. Claire's call for a philosophical dialogue targets the broader EST macrosystem, challenging the underlying institutional culture, values, and ideologies regarding an educator's duties. As the data suggests, empathy often stems from personal experience, such as having a child in elite sport, without which some might perceive students’ needs as entitlement.

Claire also emphasized the need for greater awareness of the unique challenges faced by foreign student-athletes at the University including adapting to the culture and language. This observation aligns with findings by Fuchs et al. (22), who noted that migrating student-athletes often face several challenges that may affect both academic and sporting performance and, in some cases, lead to dropout. Thus, care must be taken to provide adequate support, potentially by pairing foreign student-athletes with local ones, to prevent student-athletes from struggling unnecessarily.

However, providing adequate support may at times be time-consuming and Joe argued that *academics should also be awarded for their efforts*. Appreciation should be expressed to academics by student-athletes and by the institution itself. Brown et al. (45) state that individuals are embedded within a web of relationships and systems that shape their experiences, actions, and emotions. If we want to create supportive and thriving environments, there needs to be trust and authenticity.

James proposed that student-athletes should also be formally rewarded for the hours they dedicate to sports and other extracurricular activities. *Support and encouragement* are necessary. It is suggested that the university adopt elements of the American system by awarding academic credit for high-level participation in sport. Being given the Dean's award may also be a merited award (Claire).

While student-athletes in Ryan et al.'s (11) study often viewed the DC path as a personal choice and, thus, their responsibility, George in this study, emphasized *creating a balance* between rights and obligations, stating that if training takes up a substantial amount of time, they should consider part-time study. Noel echoed this, supporting flexibility for international competition-related absences but cautioning against compromising academic integrity, stating that student-athletes should follow the University's attendance requirements. Various authors (27, 46) have highlighted that student-athletes often choose academic programs with greater flexibility or lighter workloads to accommodate their sporting commitments. However, this may not be based solely on their academic interests, but on what allows them to succeed in both.

Luke believes that the university should increase the availability of part-time and online courses to better accommodate the needs of diverse student populations. Similarly, James noted that the introduction of hybrid/online learning made things much easier, stating: “You can now miss a lecture without any penalty, as you can attend online. This system is now integrated into our faculty's teaching structure.” The recording of lectures was also highlighted as a valuable resource for student-athletes, providing greater flexibility in managing their training schedules.

Participants in Ryan et al.'s (11) study similarly indicated that online learning options enhanced their ability to manage DCs as they could access course content during more convenient times, without needing to reschedule or miss sessions. However, other participants preferred face-to-face engagement, highlighting the value of direct interaction and personalized feedback. Consequently, a blended learning model appears to be the most effective and inclusive approach for student-athletes.

Faculties and departments should also explore various methods for managing courses. For instance, the Faculty of IT offers a Master's program structured around an intensive week of lectures, followed by approximately three months to complete the assignments (George, Noel). Isidori (47) recommends that universities implement policies addressing both curriculum content and the logistics of learning delivery. This includes developing innovative approaches that extend beyond the traditional classroom setting.

The University should also consider introducing the option for a career break (Noel, Joe). Joe proposed that rather than relying only on suspending studies, which often carry negative connotations, students could instead be offered the option of an academic career break, for whatever reason. This would allow them to pause their studies for reasons such as focusing on their sporting career, without implying illness or failure. He suggested that such breaks could be limited (e.g., up to two per student) and tied to clear outcomes to prevent misuse.

Claire also expressed concern about students who prolong their studies indefinitely. She emphasized the importance of faculty support in helping students reintegrate, noting that many never return after suspending their studies, and those who do need close follow-up, especially at the start. George expressed concern that prolonged delays in student research could render the work obsolete or redundant, as similar studies may be published in the meantime or the relevance of the topic may diminish over time.

For Owen and Jasmine, the education system is rigid. As Owen stated, “Our education system is still focusing on academic work, and only if time is left can you engage in other activities.” In line with Borggrefe and Cachay (48), universities that aim to support high-performance student-athletes effectively focus on adapting courses, study programs, and teaching methods to offer more flexible learning options to this distinct group. Entry requirements for certain programs, such as medicine, may also need to be reconsidered, for instance, the requirement to have results in a single certificate (Noel, Joe).

Greater *promotion* of the SASP should also be undertaken to attract prospective students to the University rather than other institutions (Alex). At the university level, long-term investment begins with increasing on-campus sporting activities and improving sports and academic facilities (Luke, Noel).

There is also a need for sustained *investment* in sport at the governmental and national levels to provide greater value and recognition to home-grown student-athletes (Luke andOwen stated. Owen and Kris oalso bserved that the national education system is not structured around athletes’ needs. Owen remarked that, during the Games of the Small States of Europe (GSSE), in some sports, financial incentives were provided to foreign athletes to secure medals, rather than investing in home-grown talent. In Xerri and Muscat's (5) study, it was also observed that student-athletes were frustrated with the inconsistent support they received at both the institutional and national levels. This lack of support caused student-athletes to face numerous pressures. Luke in the current study highlighted the influence of political culture on progress in this area:

Political culture plays a decisive role. I think we still need to professionalize these things. You cannot have a new person in a high-ranking position in sports with new ideas and then ignore anything that happened before. Continuity is important, and this is very symptomatic of the entire sports sector.

EU policy advocates for improved support for student-athletes (3), however, the implementation of such policies remains inconsistent (49). Employing a systems-led approach, as suggested by Wagstaff and Quartiroli (8), that focuses on psychologically informed environments in which a range of stakeholders are involved in providing appropriate support to student-athletes and those working with them is crucial.

This theme represents efforts to restore balance across HAC layers, such as:

- Inter-faculty dialogue → strengthening psychosocial and institutional coherence
- Mentoring and incentives → supporting psychological and academic development
- Promotion of DC programs → improving visibility across domains
- Balancing rights and obligations → aligning academic and athletic trajectories.

Strategies proposed above by academics reflect attempts to operationalize a more holistic DC environment by addressing multiple developmental domains simultaneously. Together, these strategies reflect a move from individualized problem-solving to system-level alignment:

- *Microsystem*: clearer communication and flexible pedagogical practices
- *Mesosystem*: structured collaboration between academic units and DC programs
- *Exosystem*: formalized policies, training, and recognition mechanisms
- *Macrosystem*: cultural shifts toward valuing sport within higher education.

Taken together, the two themes illustrate a continuum in how academics engage with DCs. While ‘Navigating Demands’ captures the tensions experienced when institutional systems and developmental domains are misaligned, ‘Cultivating a winning environment’ reflects academics’ recognition of the need for coordinated, multi-level responses. Viewed through a holistic and ecological lens, effective DC support emerges not as an individual accommodation but as a shared institutional responsibility.

## Conclusion, implications, and limitations

The findings and conclusions of this study underscore that effective dual-career support requires a shift from discretionary, individualized academic practices to coordinated, institutionally embedded systems similar to that suggested by Wagstaff and Quaritoli (8). The spectrum of academic perceptions identified, ranging from strong advocacy for recognizing student-athletes as a distinct population to concerns about equity and potential misuse of support, highlights the necessity for clear, transparent policies that ensure consistency across faculties. Consistent with the HAC model, the results demonstrate that DC challenges cannot be reduced to time management alone; they reflect interconnected academic, psychological, psychosocial, and institutional demands. Ecologically, the findings reveal that misalignment across systems—particularly weak communication and limited policy guidance—places undue responsibility on individual academics, increasing the risk of inequitable support and athlete disengagement.

At a broader level, the study reinforces the importance of culturally and structurally sensitive DC frameworks in small states such as Malta, where limited professional sport pathways and infrastructure amplify the need for educational institutions to play a central role in athlete development. The call for a national DC policy aligns with European recommendations and underscores universities’ potential to serve as key agents within a coordinated national support ecosystem.

While the study provides in-depth insights into academics’ perceptions, it is limited to a single small state, which may constrain its broader generalizability. Relying solely on academics’ perspectives excludes the views of student-athletes, coaches, and policymakers, whose inclusion could enrich understanding of system-level interactions. Additionally, divergent academic views may reflect disciplinary cultures and personal engagement with sport, which were not explored in depth. Nevertheless, these limitations do not detract from the study's contribution; rather, they point to directions for future multi-stakeholder and comparative research with other GSSE participant nations, together with an action research approach to improving support within an academic institution.

### Practical Recommendations for Universities and Policy Makers

Building on the findings and conclusions, the following recommendations are proposed:

1. Formalize DC Policies at the Institutional Level

Universities should adopt clear DC policies that define eligibility criteria, levels of support, and staff responsibilities, reducing reliance on individual discretion and ensuring equitable treatment.

2. Enhance Awareness and Communication

Internal communication strategies should ensure that academics are aware of existing DC programs, student-athlete status, and support pathways.

3. Provide Structural Flexibility

Support should move beyond *ad hoc* arrangements to include systemic measures such as recorded lectures, asynchronous learning, part-time pathways, and online provision where feasible.

4. Invest in Staff Development

Targeted training in DCs, mental health awareness, and inclusive pedagogical practices is essential to support academics in managing complex DC needs.

5. Ensure Academic Buy-in and Co-design

Academics should be actively involved in the design, implementation, and evaluation of DC support programs to ensure feasibility, fairness, and sustainability.

6. Establish Central Coordination Units

Each institution should designate a DC coordinator or unit to liaise with faculties, coaches, and national sport bodies, minimizing administrative burden on academics.

7. Develop National DC Guidelines

In line with FEPSAC (20) recommendations, a national, tiered DC framework should be developed to support student-athletes most in need while maintaining academic integrity, particularly relevant in small-state contexts.

These recommendations are highly transferable to other small states or resource-limited universities under three conditions: (1) limited budget; (2) need for structure; The campus currently relies on informal agreements that need to be turned into an official policy; (3) cultural readiness: The institution views supporting student-athletes as a core educational duty rather than a favor.

Together, the findings, implications, and recommendations reinforce the conclusion that sustainable DC support depends on coordinated, holistic, and policy-driven approaches that recognize academics as essential partners within an integrated DC ecosystem.

## Data Availability

The datasets presented in this article are not readily available because participants did not consent to sharing interview transcripts. Requests to access the datasets should be directed to the corresponding author.
