# Supplementary material for: Two worlds, one goal: a qualitative study of academics’ perceptions of student-athletes
Source: Front Sports Act Living. 2026 Jul 14;8:1835408. doi: 10.3389/fspor.2026.1835408 (PMC13410515; doi:10.3389/fspor.2026.1835408)
Supplement: Supplementary file 1 [file Supplementaryfile1.docx]

**Exploring the perceptions of academics on dual-careers**

**Introductory questions + explanation of research**

- 1. Approximately how long have you been an academic at UOM? During this time, on average, yearly (or over a longer defined period) how many student-athletes have you come across in your department/faculties?
- 2. What do you understand by the term dual career or student-athlete?

**Academic Roles**

- 3. Are you a student-athlete mentor? If yes, what is your role? If no, what do you think a mentor’s role should be for student-athletes?
- 4. What is your personal opinion on students-athletes following a dual career? Give reasons for your answers.
- How many hours do you think student-athletes spend weekly on their training and on their academic work?
- 5. What do you believe are the benefits derived by a student-athlete in following a dual-career?
- 6. What are the challenges they may face? (academically, personally, socially etc.)

**Support and Personality Traits**

- 7. What do you understand by the term academic flexibility for student-athletes?
- 8. Does your department/faculty support student-athletes? In what way?
- 9. What challenges does your department/faculty face in supporting student-athletes? Any particular stories/examples you would like to share?
- 10. Do you personally support student-athletes? If yes in what ways? If not, why not? Would you follow their sporting achievements, in what way?
- 11. Are you aware of any support systems for athletes at the University and even outside of this institution? Discuss.
- 12. Do you believe other students are supportive or not towards student-athletes? In what way?
- 13. In your experience, do student-athletes on average perform better academically or worse than students who are not athletes? What do you believe are the reasons for your answer? Also, have you recognised any particular traits student-athletes have in comparison to other students (eg. Perfectionism; competitiveness)?

**Conclusion**

- 14. How do we support other interests outside academic work?
- 15. Any concluding thoughts on the subject?
